# Treatment of Advanced NSCLC Patients with an Anti-Idiotypic NeuGcGM3-Based Vaccine: Immune Correlates in Long-Term Survivors

**DOI:** 10.3390/biomedicines13051122

**Published:** 2025-05-06

**Authors:** Zaima Mazorra, Haslen H. Cáceres-Lavernia, Elia Nenínger-Vinageras, Leslie M. Varona-Rodríguez, Carmen Elena Viada, Zuyen González, Nely Rodríguez-Zhurbenko, Anne-Christine Thierry, Gisela María Suarez-Formigo, Yendry Ventura-Carmenate, Petra Baumgaertner, Sara Trabanelli, Camila Jandus, Tania Crombet

**Affiliations:** 1 Center of Molecular Immunology, Havana 11600, Cuba; carmen@cim.sld.cu (C.E.V.); zuyen@cim.sld.cu (Z.G.); nely@cim.sld.cu (N.R.-Z.); taniac@cim.sld.cu (T.C.); 2 Department of Immunology, Abu Dhabi Stem Cells Center (ADSCC), Abu Dhabi 23231, United Arab Emiratesyendry.ventura@adscc.ae (Y.V.-C.); 3 Clinical Oncology Department, University Hospital “Hermanos Ameijeiras”, Havana 10400, Cuba; hassiul1978@gmail.com (H.H.C.-L.); nenin@infomed.sld.cu (E.N.-V.); lmagdielv@gmail.com (L.M.V.-R.); 4 Center of Experimental Therapeutics, Department of Oncology, Lausanne University Hospital (CHUV), 1005 Lausanne, Switzerland; anne-christine.thierry@chuv.ch (A.-C.T.); petra.baumgartner@hospvd.ch (P.B.); 5 Department of Pathology and Immunology, University of Geneva, 1211 Geneva, Switzerland; sara.trabanelli@gmail.com (S.T.);; 6 Ludwig Institute for Cancer Research, Lausanne Branch, 1066 Lausanne, Switzerland; 7 Geneva Center for Inflammation Research, 1211 Geneva, Switzerland; 8 Translational Research Center for Oncohematology, Department of Medicine, Faculty of Medicine, University of Geneva, 1211 Geneva, Switzerland

**Keywords:** non-small cell lung cancer, anti-idiotypic cancer vaccine, circulating biomarkers

## Abstract

**Background:** Racotumomab-alum is an anti-idiotype vaccine targeting the NeuGcGM3 tumor-associated ganglioside. Clinical trials in advanced cancer patients have demonstrated low toxicity, high immunogenicity and clinical benefit. The goal of this study was to identify circulating biomarkers of clinical outcome. **Methods:** Eighteen patients with stage IIIb/IV non-small-cell lung cancer (NSCLC) were injected with racotumomab-alum as switch maintenance therapy after first-line chemotherapy. Treatment was administered until severe performance status worsening or toxicity. The frequencies of innate and adaptive lymphocytes were assessed by flow cytometry. Circulating factors were measured using multi-analyte flow assay kits. **Results:** The median overall survival was 16.5 months. Twenty-seven percent of patients were classified as long-term survivors. Patients with lower baseline frequencies of CD4+Tregs and central memory (CM) CD8+T cells displayed longer survival rates. Furthermore, higher baseline frequencies of NKT cells and a high CD8+T/CD4+Treg ratio were associated with longer survival. Interestingly, patients with significantly lower levels of effector memory (EM) CD8+T cells survived longer. The levels of NKT cells and terminal effector memory (EMRA) CD8+T cells were higher in long-term survivors in comparison with short-term survivors in post-immune samples. As expected, the ratio of CD8+T/CD4+Tregs showed significantly higher values during treatment in patients with clinical benefits. Regarding serum factors, pro-tumorigenic cytokines significantly increased during treatment in poor survivors. **Conclusions**: In advanced NSCLC patients receiving racotumomab-alum vaccine, longer survival could be associated with a unique profile of circulating lymphocyte subsets at baseline and during treatment. Additionally, certain pro-tumor-related cytokines increased in short-term survivors. These results should be confirmed in larger randomized clinical trials. This clinical trial was registered in the Cuban Clinical Trials Register (RPCE00000279).

## 1. Introduction

Lung cancer remains the leading cause of cancer-related deaths worldwide. Non-small cell lung cancer (NSCLC) is a subtype of the most frequently diagnosed cancer in the world. Its epidemiology depends not only on tobacco exposure, but also on air quality [1]. According to epidemiological studies, Cuba shows one of the highest incidence and mortality rates of lung cancer in both males and females in the Latin American region [2].

The prognosis of lung cancer is challenging since most cases are diagnosed in the late stages when surgery is no longer a viable option because of distant metastases [3]. Over the last years, crucial improvements have been achieved in treatments for NSCLC, mainly due to the development of targeted therapies and immunotherapy. This latter approach includes therapeutic vaccines, immune modulators, autologous cellular therapies, and monoclonal antibodies (MAbs) directed against checkpoint inhibitor signals associated with activated T cells and/or cancer cells [4].

Therapeutic cancer vaccines aim to promote antigen-specific immune responses by presenting tumor-associated antigens (TAAs) to the patient’s immune system in the cancer environment. These vaccines face multiple challenges such as the selection of appropriate antigen and adjuvant, as well as the ability to overcome the immunosuppressive tumor environment [5].

One of the attractive targets used in cancer vaccines are the Neu-glycolyl (NeuGc)-containing gangliosides, particularly the NeuGcGM3 antigen. The expression of these molecules is associated with tumor malignancy, invasiveness and metastasis [6,7]. Low levels of NeuGc-containing molecules have been detected in healthy human tissues due to dietary acquisition [8]. Notably, the detection of elevated NeuGc levels has been repeatedly found in tumor cells and serum samples from cancer patients [9]. In addition to its exogenous incorporation in cancer cells, recent findings support the hypothesis of the de novo biosynthesis of these molecules in human cancer cells under certain metabolic conditions [10].

The racotumomab-alum vaccine is an anti-idiotypic vaccine able to mimic the ganglioside NeuGcGM3. It was developed at the Center of Molecular Immunology, in Havana, Cuba. In a preclinical model of Lewis lung carcinoma, this vaccine showed a significant reduction of spontaneous lung metastases that was associated with an increase in the number of T cells infiltrating the metastases [11]. Different phase I clinical trials and compassionate use studies have demonstrated low toxicity and encouraging clinical results [12,13]. A phase II/III randomized double-blind clinical trial in advanced NSCLC patients showed a significant improvement in overall survival (OS) and progression-free survival for racotumomab-alum versus placebo [14]. Based on these clinical results, racotumomab-alum (commercial name: Vaxira) received conditioned registration in 2013 and final product registration in Cuba in 2021 for advanced NSCLC patients [15]. Recently, the use of this vaccine in routine clinical practice prolonged the overall survival in patients with NSCLC treated in switch maintenance, and in stage IV patients who received the treatment as a second-line therapy [16]. Interestingly, an analysis of survival data from randomized clinical trials revealed a bimodal distribution, indicating the existence of two distinct patient populations following treatment with this vaccine. These populations were classified according to the clinical benefit in short- and long-term survivors. The vaccine showed greater benefits for the patients belonging to the subpopulation of long-term survivors (median OS: 76.6 months) as compared to long-term survivors treated only with conventional chemo-radiotherapy (median OS: 33.8 months) [17].

The mechanism underlying the efficacy of this vaccine and the potential biomarkers of clinical benefit are poorly understood. The present work was set up to determine, for the first time, whether specific subsets of peripheral immune cells and circulating factors might be related to long-term survival in advanced NSCLC treated with the racotumomab-alum vaccine. Based on our findings, longer survival seems to be associated with the frequencies of regulatory CD4+T cells (Tregs), CD8+T lymphocytes subsets and NKT cells in peripheral blood mononuclear cells (PBMCs) at baseline and during treatment. Certain pro-tumor-related cytokines increased in short-term survivors.

## 2. Materials and Methods

### 2.1. Patients and Treatment

**Figure 1:**
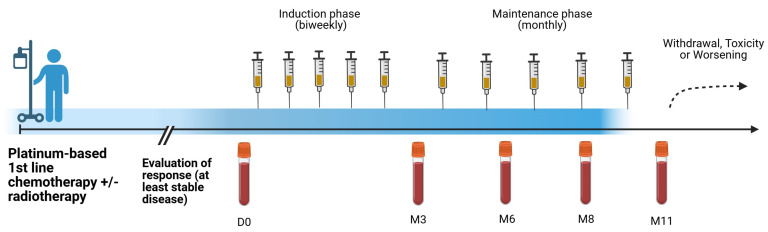
Immunization and sampling schedule of NSCLC patients treated with racotumomab-alum vaccine. Created with BioRender.com.

This was a post-marketing exploratory study in 18 histo- or cytologically confirmed heavily-treated advanced NSCLC patients (RPCE00000279). The subjects were injected intradermally with the NeuGcGM3 anti-idiotype vaccine (racotumomab-alum) consisting of 1 mg aluminum hydroxide-precipitated murine racotumomab MAb as switch maintenance therapy. The first five doses were administered every 14 days, and the remaining 10 doses were administered every 28 days. After 15 doses, reimmunizations were administered at 28-day intervals if the patients maintained a favorable clinical status (Figure 1).

The study was carried out in compliance with the Helsinki Declaration. The protocol was approved by the ethics committee of “Hermanos Ameijeiras” University Hospital in Cuba (25 October 2017). All patients were required to sign a written informed consent before enrollment into the study.

Regarding clinical outcome, patients who lived 24 months or more after vaccination were classified as long-term survivors. All of the rest were considered short-term survivors.

### 2.2. Safety

All patients included in the study were evaluated for safety. The frequency, nature, causality and severity of adverse events were evaluated. Severity was graded according to the NCI Common Toxicity Criteria for Adverse Events (version 4.0). Laboratory assessments were performed during the vaccine administration period.

### 2.3. Collection of Peripheral Blood Mononuclear Cells and Sera

PBMCs and sera were collected at baseline and after 3, 6, 8 and 11 months of treatment. Briefly, from 20 mL of blood, the mononuclear fraction was isolated by Ficoll-Hypaque density gradient separation, washed three times with phosphate buffered saline (PBS), and cryopreserved in 90% heat-inactivated fetal calf serum (FCS) (Gibco, MT, USA) and 10% DMSO in liquid nitrogen at a concentration of 1 × 10^7^ cells/mL until assayed. An additional 5 mL of blood was used for serum isolation.

### 2.4. Flow Cytometry

Multi-color flow cytometry analysis was performed on thawed PBMCs from referred time points. Each panel was optimized to ensure minimal spectral overlap among fluorochromes. Between 1 and 3 million viable PBMCs per patient were stained for each panel. Three healthy donor PBMCs, isolated and cryopreserved according to the aforementioned procedures, were included per run to ensure that inter-run variability remained below 10%. PBMCs were washed with PBS and incubated with LIVE/DEAD Fixable Zombie green or Zombie NIR for 20 min at 4 °C. PBMCs were washed and stained with a cocktail of fluorescently labeled antibodies for 30 min at room temperature (RT) in the dark. Subpopulations of αβ T cells, γδ T cells, NK, NKT and innate lymphoid cells (ILCs) were analyzed. The MAbs used for staining are depicted in the following table (Table 1).

Cells were washed 3 times and analyzed on a LSRFortessa^TM^ cell analyser (BD Biosciences, San Jose, CA, USA) using FACSDiva v7.0 software. Application settings for each panel were used for all data acquisition. Dead cells, doublets and debris were removed from FCS files prior to analysis using FlowJo-v10 software (Tree Star Inc., Ashland, OR, USA).

### 2.5. Multiplex Assays for Soluble Factors

The LEGENDplex™ Human Immune Checkpoint Panel 1 (12-plex) (740867, BioLegend, London, UK) and LEGENDplex™ Human Cytokine Panel 2 (13-plex) (740102, BioLegend, London, UK) were used to quantify serum cytokines and immune checkpoint-related proteins at baseline and during racotumomab-alum treatment. Assays were performed according to the manufacturer’s instruction. Briefly, sera were mixed with specific antibody-coated beads that promote the formation of an analyte–antibody complex. After washing, biotinylated detection antibodies were added to bind to the specific analyte adsorbed to the capture beads, thus forming capture bead-analyte-detection antibody sandwiches. The addition of streptavidin–phycoerythrin, which binds to the biotinylated detection antibodies, provides fluorescent signal intensities that are proportional to the amount of bound analyte. Fluorescent signals were measured by dual-laser flow cytometry (Attune NxT) and analyzed using LEGENDPLEX^TM^ data analysis v8.0 software.

### 2.6. Statistical Analyses

The Shapiro–Wilk normality test was used to determine the normal distribution of variables. Statistical differences in immune cell population frequencies and soluble factor concentrations between the short- and long-term survivors at different time points were evaluated using the nonparametric Mann–Whitney test. The Wilcoxon Signed-Ranks Test for matched pairs was used to compare patients’ samples before and after treatment. Overall survival was calculated as the period between the start of vaccination and the date of death, or the last follow-up. Survival data were analyzed using the Kaplan–Meier method and the log-rank test was applied to explore the differences in OS between short- and long-term survivors and in relation to immunological variables. Cutoff points for survival analysis were determined using Cutoff Finder v1.0 software.

The graphs and referred analysis were performed in GraphPad Prism v8.0 software. Statistical analyses were made with SSPS program (version 16.0). The signification level was assumed as 0.05 for all of the hypotheses tested.

## 3. Results

### 3.1. Patient Characteristics and Treatment Outcome

Eighteen advanced NSCLC patients were included in this study. Detailed information can be found in Table 2. All patients were in stage III or IV, and 94% had a performance status (PS) ≤ 1 prior to entering the study. Sixteen patients reached stable disease (SD) or better at least 4 weeks prior to the inclusion in the trial. Most of these patients had received more than one line of chemotherapy combined with radiotherapy before racotumomab-alum treatment. Two patients were progressors and unfit for second-line cytotoxic treatment.

**Figure 2:**
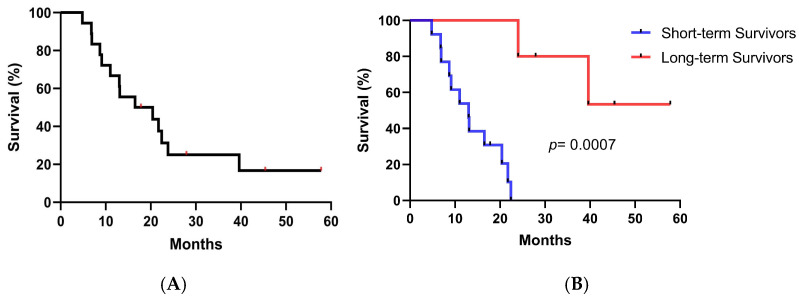
Overall survival. (**A**) Kaplan–Meier curve for OS in months for all patients (*n* = 18), calculated as the difference between the vaccination onset and the date of death (*n* = 14), or date of last visit (*n* = 4). (**B**) Kaplan–Meier curves for short- and long-term survivors. Differences in survival times were assessed by the log-rank test. Red spots (**A**) and black spots (**B**) represent censored patients.

The median overall survival (MOS) was 16.5 months (95% confidence limits: 3.78–29.21) (Figure 2A). To better interpret the data, patients were classified into long-term survivors (≥24 months) and short-term survivors (<24 months). In this case, five out of eighteen (27.8%) were long-term survivors. The median survival time was not reached in long-term survivors while it was 13 months in the short-term survival group (*p* = 0.0007, log-rank test) (Figure 2B).

### 3.2. Safety

The adverse events reported in this study were very similar to the ones previously published for the clinical trials using the racotumomab-alum vaccine [18]. The toxicity was classified as grade 1 and 2, according to the NCI Common Toxicity Criteria (version 4.03). The most common adverse events were local reaction at the injection site with erythema and induration occasionally associated with mild pain that lasted for a few days (1–3 days). Neither biochemical nor hematological abnormalities were reported.

### 3.3. Changes in Immune Cell Populations in Short-Term and Long-Term NSCLC Survivors Treated with Racotumomab-Alum Vaccine

**Figure 3:**
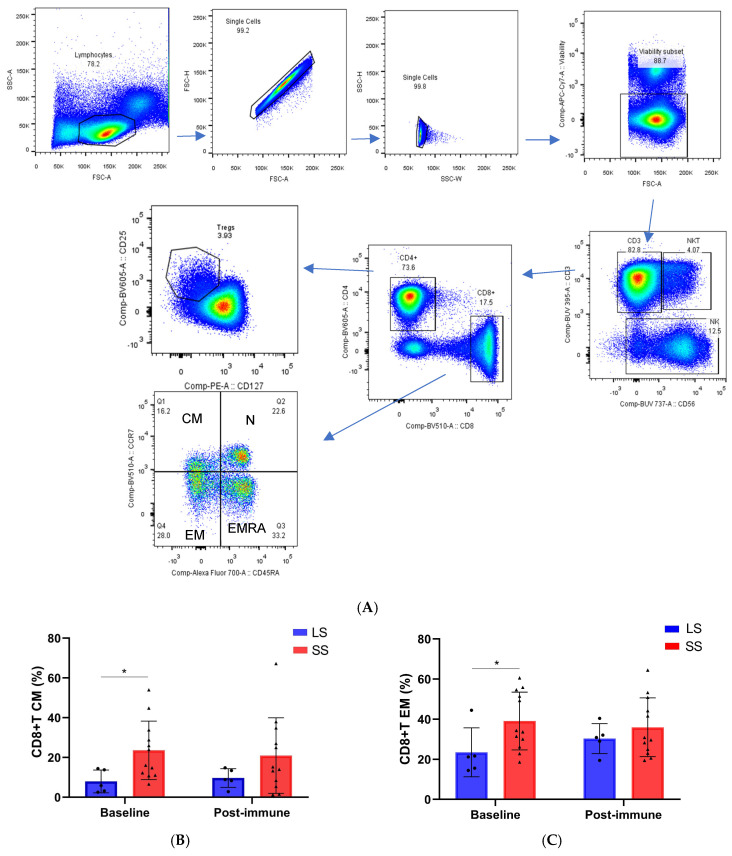

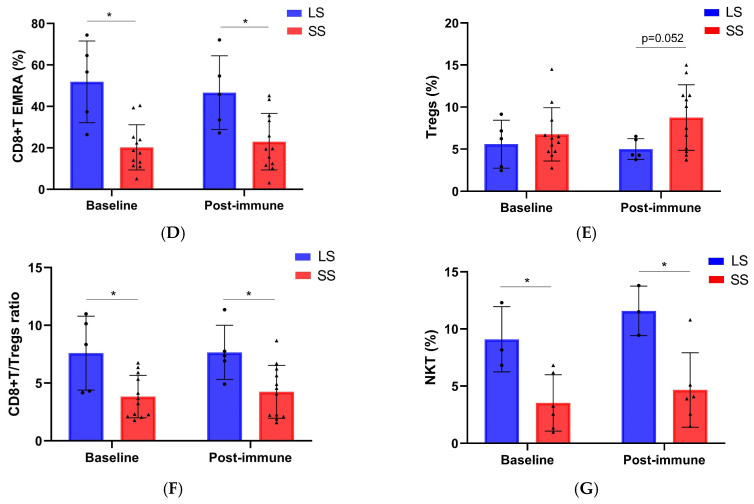
Changes in immune cell populations during racotumomab-alum treatment in long-term (LS) and short-term survivors (SS). (**A**) gating strategy to analyze NK, NKT and T cell subsets is shown. Lymphocytes were subjected to doublet discrimination. Singlet lymphocytes were separated according CD3 and CD56 expression to identify NK (CD56+CD3−), NKT (CD56+CD3+) and T cells (CD56−CD3+). T cells were plotted using CD4 and CD8 to further divide the population. CD8+T cells were further resolved according to CCR7 and CD45RA expression: CCR7+CD45RA+ as naïve (N), CCR7+CD45RA− as central memory (CM), CCR7−CD45RA− as effector memory (EM) and CCR7−CD45RA+ as terminal effector memory T cells (EMRA). CD4+T cells were plotted for CD25 and CD127 to identify Tregs as CD25+CD127−. (**B**–**D**) Behavior of CD8+T cell subpopulations, (**E**) and (**F**) CD4+Tregs cells and CD8+T/CD4+Tregs cells ratio and (**G**) NKT lymphocytes in long- and short-term survivors during racotumomab-alum vaccination. The black triangles and circles show the values for each patient. The asterisks indicate statistically significant differences between the groups (* *p* < 0.05, Mann–Whitney *U* test).

The frequencies of circulating T cell subpopulations were assessed at baseline and after 6–8 months (post-immune samples) from baseline in 17 NSCLC patients treated with the racotumomab-alum vaccine and with available pre- and post-immunization samples. Gating strategies for maturation stages of CD8+T cells (CCR7+CD45RA+ naïve, CCR7+CD45RA− central memory (CM), CCR7−CD45RA− effector memory (EM) and CCR7−CD45RA+ terminal effector memory cells (TEMRA)) and for regulatory CD4+T cells (CD4+CD127−CD25hi) are displayed in Figure 3A. The comparison of circulating lymphocyte subsets frequencies between long-term survivors (≥24 months) and short-term survivors (<24 months) at baseline and during treatment is shown in Figure 3B–G. Both CM and EM CD8+T cells showed higher frequency in short-term survivors at baseline as compared to long-term survivors. On the contrary, long-term survivors had higher frequency of EMRA CD8+T cells at baseline and this difference persisted after 6 to 8 months of treatment (Figure 3B–D). Regarding CD4+Tregs cell frequency, no differences were found at baseline between these two groups of patients. Furthermore, a trend to a higher frequency of Tregs cells was detected during treatment in patients with short-term survival (Figure 3E). Notably, the ratio of CD8+T/CD4+Treg cells was significantly higher in long-term survivors at baseline and after 6–8 months of treatment in comparison with short-term survivors (Figure 3F). No differences in the frequency of CD4+T cell subsets between long- and short-term survivors were detected (Supplementary Figure S1). 

Regarding innate lymphocytes, the frequencies of γδ T cells (CD3+γδTCR), NK (CD3−CD56brightCD16− or CD3-CD56dimCD16+), NKT (CD3+CD56+), innate lymphoid cells (ILCs) (Lin-CD127+) and their subpopulations, were measured in nine patients with available samples at baseline and during racotumomab-alum treatment. The frequency of NKT cells was significantly higher in long-term survivors at baseline and during treatment as compared to short-term survivors. (Figure 3G). No differences in the frequencies of the rest of innate lymphocytes were found (Supplementary Figure S2). The gating strategy of CD4+T cells and innate lymphocytes is shown in Supplementary Figure S3. No significant differences in the frequencies of immune subsets between baseline and post-immune samples were found (Supplementary Figure S4). The frequencies (%) of the populations from Figure 3 are shown in Supplementary Table S1.

### 3.4. Changes in Circulating Factor Levels in NSCLC Patients During Racotumomab-Alum Vaccine Treatment

**Figure 4:**
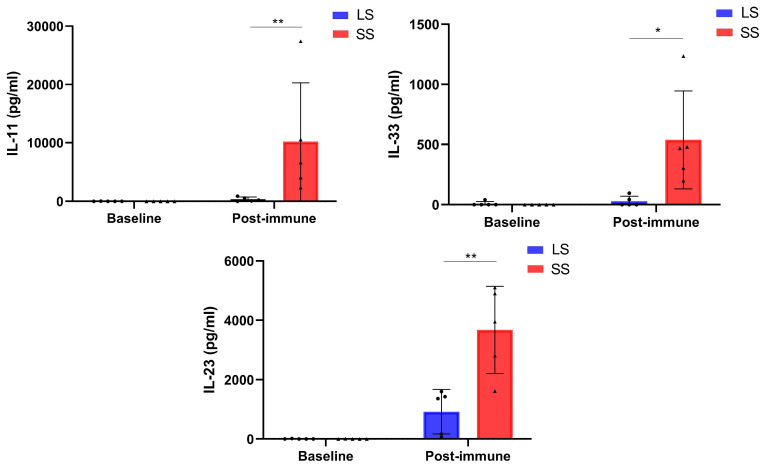
Changes in serum cytokine levels in long-term (LS) and short-term survivors (SS) at baseline and at post-immune time points (3–6 months). The black triangles and circles show the values for each patient The asterisks indicate statistically significant differences between the groups (* *p* < 0.05 and ** *p* < 0.01, Mann–Whitney U test).

We assessed the levels of 12 oncology-related immune checkpoint biomarkers and 13 inflammatory cytokines in the sera of 10 NSCLC patients with available samples at baseline and during racotumomab-alum treatment. In short-term survivors, median serum levels of the pro-tumorigenic interleukins (IL)-11, IL-23 and IL-33 at post-immune time points (3–6 months) after racotumomab-alum onset were significantly higher when compared with the values observed in long-term survivors at the same time points (Figure 4). There were not statistically significant differences in the above-mentioned cytokine concentrations at baseline between short- and long-term survivors. The levels of cytokines are shown in Supplementary Table S2.

### 3.5. Associations Between Immune Cell Populations at Baseline and Overall Survival

**Figure 5:**
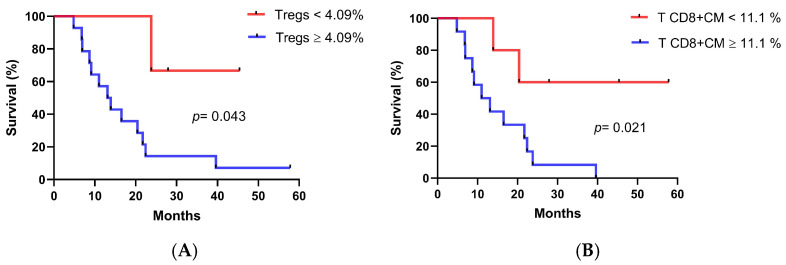

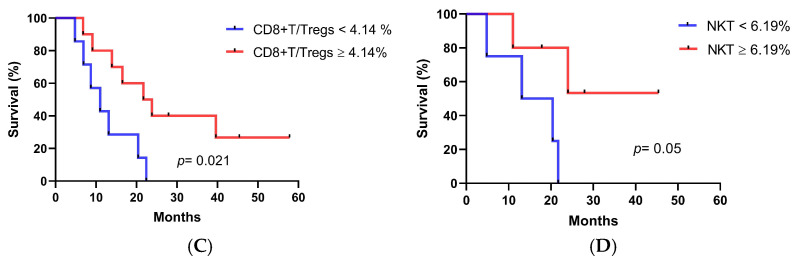
Association of immune cell populations at baseline with overall survival. Kaplan–Meier curves representing overall survival vs. immune cell subsets after dichotomizing the data at established cut off. Patients were divided according to the percentage of (**A**) CD4+Tregs cells, (**B**) CM CD8+T cells, (**C**) CD8+T/Tregs cells ratio and (**D**) NKT cells. Differences in survival times were assessed by the log-rank test.

Analyses of baseline immune cell population values were performed to identify those subsets associated with longer OS. In this case, T cell populations from seventeen patients and innate lymphocytes from nine patients were analyzed. As seen in Figure 5A,B, patients with a lower percentage of CD4+Tregs cells (<4.09%) and CM CD8+T cells (<11.1%) at baseline displayed longer OS (CD4+Tregs cells, medians: 13.1 months vs. not reached *p* = 0.043, log-rank test; CM CD8+T cells, medians: 11 months vs. not reached *p* = 0.021, log-rank test). As expected, patients with a higher CD8+T/CD4+Tregs ratio (4.14) at baseline survived significantly longer (CD8+T/CD4+Tregs cells ratio, medians: 11 months vs. 21.7 months *p* = 0.021, log-rank test) (Figure 5C). Interestingly, a higher frequency of NKT cells (≥6.19%) correlated with longer OS (NKT cells, medians: 13.1 months vs. not reached *p* = 0.050, log-rank test) (Figure 5D).

## 4. Discussion

Different studies have established the definition of long-term survival in advanced NSCLC at more than 2 years from the time of diagnosis, with rates of survivorship after one or more lines of therapy ranging from 8 to 16%. Most of these studies include treatment with immune checkpoint inhibitors (ICI) [19,20,21]. Due to the heterogeneous duration of conventional treatment periods, our study used a more stringent classification of long-term surviving patients taking into account the onset of vaccination instead of diagnosis date. The 2-year OS rate for patients who received the racotumomab-alum vaccine as switch maintenance therapy was 27.8% (five out of eighteen). This value is similar to what has been reported for ICI treatments. A recent updated analysis of the KEYNOTE-010 study showed 2-year OS rates of 14.5% for docetaxel versus 30.1% and 37.5% for pembrolizumab 2 mg/kg and 10 mg/kg every 3 weeks, respectively [22]. Similarly, in the OAK trial, patients treated with atezolizumab displayed a 2-year OS rate of 28% [23]. In the case of nivolumab-treated patients, pooled data from CheckMate studies showed OS rates of 26.9% as compared to 13.5% in the docetaxel group. The median OS was 11.1 months for nivolumab-treated patients as compared to 8.1 months for those treated with docetaxel [24].

The median OS of patients from the present study was 16.5 months. Although this is a small series of patients, the results are encouraging, considering that they were biomarker-unselected, heavily-treated advanced cancer patients. Previously, in a phase III clinical trial using an EGF-based vaccine (CIMAvax), vaccinated patients showed a median OS of 10.83 months. In the biomarker-based analysis, those patients with high baseline EGF serum levels had 14.66 months as the median OS, compared with 8.63 months for those with similar EGF serum concentrations who were not vaccinated [25]. The CIMAvax-treated patients received only front-line chemotherapy before immunotherapy administration. They were included in the vaccination treatment if they reached at least stable disease after platinum-based first-line chemotherapy. In contrast, the patients in our study received different chemotherapy lines before the initiation of vaccination. Interestingly, it was previously shown that the survival analysis of advanced NSCLC patients treated with racotumomab-alum vaccine was adjusted to a bimodal distribution. In this case, 19% of patients were classified as long-term survivors. These patients benefited remarkably from the treatment, since the median OS was 76.6 months [17].

This study aimed to determine changes in immune subpopulations and soluble factors during racotumomab-alum treatment, as well as to identity potential baseline biomarkers associated with clinical outcomes. We compared the immune profiles of patients with long- and short-term survivals. In this sense, previous publications have shown that increased numbers of blood and intra-tumoral Tregs correlated with worse prognosis and a higher risk or recurrence in patients with NSCLC [26,27]. Moreover, significantly higher percentages of CD4+CD25+FoxP3+Tregs have been observed in patients with advanced metastatic NSCLC compared to healthy donors [28]. In our study, patients with a higher frequency of Tregs at baseline survived for a shorter time. In addition, these frequencies remained high after racotumomab-alum vaccination in patients with short survival. In long-term survivors, the levels of Tregs did not increase after treatment, remaining at the baseline level. Controversial results have been found regarding regulatory T cells and clinical outcome in cancer patients treated with immunotherapies. Previous studies have reported that high frequencies of circulating Tregs after anti-PD-1 immunotherapy have been associated with a favorable clinical outcome in advanced lung cancer patients [29,30]. In contrast, other studies reported that lower levels of Tregs before ICI treatments in NSCLC patients were associated with better tumor response [31,32]. In addition, several preclinical and clinical reports support the notion that the elimination of Tregs is crucial for the effectiveness of various cancer therapies [33,34]. Furthermore, we found that a high effector/suppressor ratio measured as CD8+T cells/CD4+Tregs is found in long-surviving patients at baseline and during racotumomab treatment. It is well known that CD8+T cells can expand and differentiate into cytotoxic T lymphocytes (CTL) that infiltrate tumors through peripheral blood migration and play an important role in antitumor immunity through the direct killing of tumor cells [35]. In line with our findings, recent papers reported that lung cancer patients with a high CD8+T cells/CD4+Tregs ratio at tumor baseline showed prolonged OS after PD-1/PD-L1 blockade [36,37]. 

Circulating and lymph node-resident CD8+T cells are classically subdivided according to their state of differentiation into naive T cells, effector T cells and subsets of memory T cells [38]. Previously, it was reported that a high circulating CM/EM ratio is associated with tumor inflammation in melanoma and lung cancer. Additionally, high CM/EM T cell ratios are associated with longer survival in NSCLC patients who received nivolumab [39]. In contrast, we found lower CM and EM CD8+T cell frequencies at baseline in long-term as compared to short-term survivors. Notably, patients with a high proportion of EMRA CD8+T cells are in the group of longer survival subjects. EMRA CD8+T cells are reported as effector memory CD45RA re-expressing T cells. They are considered a terminally differentiated subset and exhibit low proliferation capacity and differentiation plasticity, while possessing an increased production of perforin and granzyme B [40]. Previous studies have identified differences in EMRA CD8+T cell phenotypes between peripheral blood and tumor sites in NSCLC patients. Specifically, it was observed that highly immunogenic tumors had a higher proportion of EMRA CD8+T cells that were negative for CD27/CD28 expression at the tumor site. In the periphery, most of the EMRA CD8+T cells had the above-mentioned phenotype and patients with low CD27/CD28 expression on these cells survived longer when treated with ICI therapies [41]. Notably, a study in long-term survival colon cancer patients vaccinated with a viral replicon-based cancer vaccine showed that higher EMRA CD8+T cells and lower Treg proportions were associated with longer survival times [42]. These findings are in line with our results, and pave the way to more deeply characterize the phenotype of CD8+T cells both in periphery and tumor sites, providing a potential use of these subsets as predictive biomarkers in racotumomab-alum vaccinated patients.

NKT cells are a heterogeneous subpopulation that exhibits the co-expression of characteristics of both conventional T lymphocytes (αβTCR, CD3) and NK cell surface markers (CD56 and CD161) [43]. In contrast to conventional T lymphocytes, NKT are able to recognize lipid or glycolipid antigens presented in the context of the non-classical antigen-presenting molecule CD1d [44]. Inside the NKT cell population, invariant NKT (iNKT) cells harbor an invariant αβ TCR (Vα24β11 in humans) and are characterized by a rapid response upon stimulation and in antitumor responses [45]. Most publications have focused on this subpopulation and have reported an increased number of iNKT at the tumor site as compared to periphery in cancer patients [46,47]. In addition, the accumulation of iNKT cells in the tumor has been correlated with a better prognosis in colon carcinoma patients [48]. Interestingly, we found a higher percentage of peripheral NKT cells in long-term survivors as compared to short-term survivors both at baseline and during racotumomab-alum vaccination. However, we detected a significantly lower percentage of iNKT in the baseline samples from cancer patients, regardless of the survival, in comparison with healthy donors (manuscript in preparation). These findings are in line with previous publications, in which numerical and functional iNKT deficiencies have been reported in cancer patients including NSCLC [49,50]. In our case, the racotumomab-alum vaccine aims at achieving an immune response against the ganglioside NeuGcGM3, which is presented in the context of CD1d [51]. There is a direct association between CD1d expression and survival in NSCLC patients [52]. Further assessments in racotumomab-alum-treated patients should consider the characterization of these cells and of the CD1d expression at the tumor site.

Circulating proteins sequentially assessed in blood samples have demonstrated potential utility in monitoring clinical responses in NSCLC patients undergoing anti-PD-1 treatments [53,54,55]. In the present study, checkpoint-related biomarkers and inflammatory cytokines were tested before and after racotumomab-alum treatment. No significant differences were found between long-term and short-term survivors regarding checkpoint-related molecules. However, our multiplex serum analyses revealed that patients classified into the poor outcome group exhibited significantly increased levels of the pro-tumoral cytokines, IL-11, lL-23 and IL-33, after vaccination. Several findings are associating high levels of these cytokines with lung tumor progression. IL-11 is a pleiotropic cytokine belonging to the IL-6-related cytokine family that has recently emerged as a tumor-promoting biomarker [56]. High IL-11 expression has been associated with poorer survival [57]. Additionally, IL-11 facilitates lung cancer cell chemoresistance via the IL-11R/STAT3 signaling pathway, which promotes the activation of anti-apoptotic proteins [58]. IL-23, a member of the pro-inflammatory cytokine family that includes IL-12, plays an important role in promoting the proliferation and effector functions of Th17 cells [59]. Previous data support the effect of endogenous IL-23 in tumor and metastasis development [60]. It is found to be overexpressed in many human tumors including lung malignancies [61]. High levels of IL-23 and sIL-23R have been associated with a lower survival rate in patients with NSCLC [62]. In the case of IL-33, it was identified as a member of the IL-1 family. It plays a dual role, either promoting or suppressing lung cancer [63]. Clinical studies show that the high expression of IL-33 in paracancerous tissues of NSCLC patients, along with elevated serum levels, correlates with increased tumor malignancy and poor prognosis [63,64]. Interestingly, IL-33 remodels the tumor microenvironment by orchestrating the development and maintenance of immune suppressive cells including Tregs, thereby allowing tumor progression [65,66]. This is in line with our findings, in which short-term survivors are characterized by increased levels of IL-33 and Tregs in the periphery. In general, much evidence points to the important role of these cytokines in lung cancer progression. Combining the blocking of the signaling pathways of these cytokines with racotumomab-alum treatment could increase the number of patients benefiting from these therapies.

This study has some limitations that warrant consideration. On one hand, these biomarkers were studied in a limited number of treated patients. On the other hand, the frequencies of these populations were not evaluated in a control group without maintenance treatment or receiving a different established therapy, limiting the role of these biomarkers as predictors of clinical response. Further immune-monitoring studies must be carried out to improve the immunological characterization of cancer patients before and during racotumomab-alum therapy, and provide oncologists with new parameters that can support the optimal choice of therapy. Currently, a randomized clinical trial is being designed to validate these populations as predictive biomarkers of clinical outcome.

## 5. Conclusions

This study presents findings indicating that longer survivals in advanced NSCLC patients treated with racotumomab-alum vaccine could be associated with a unique profile of peripheral lymphocyte subpopulations at baseline and throughout the vaccination period. Additionally, tumor progression-associated cytokines seem to increase in short-term survivors. These biomarkers tested in blood might potentially be used to monitor clinical response in NSCLC patients treated with this vaccine, or targeted in combination to increase efficacy. Nonetheless, this study is constrained by its small sample size which restricts the statistical power necessary for comprehensive analysis. Despite this limitation, the identification of significant associations within such a small cohort is encouraging. Consequently, these results should be considered as hypothesis generating and warrant further investigation in larger, randomized immunotherapy trials, including a control group of patients without this vaccine as maintenance treatment.

## Figures and Tables

**Table 1:** Anti-human antibodies used for flow cytometry.

Antibody	Fluorophore	Clone	Catalogue Number/Company	Dilution
Anti-CD3	FITC	UCHT1	300440/Biolegend	1/100
Anti-CD4	FITC	RPA-T4	300515/Biolegend	1/200
Anti-CD8	FITC	RPA-T8	301006/Biolegend	1/100
Anti-CD14	FITC	HCD14	325604/Biolegend	0.3/50
Anti-CD15	FITC	HI98	301904/Biolegend	0.3/50
Anti-CD16	FITC	3G8	302006/Biolegend	1/400
Anti-CD19	FITC	HIB19	302206/Biolegend	1/400
Anti-CD20	FITC	2H7	302304/Biolegend	0.3/50
Anti-CD33	FITC	HIM3-4	303304/Biolegend	1/200
Anti-CD34	FITC	581	560942/BD Pharmingen	1/100
Anti-CD203c	FITC	NP4D6	324614/Biolegend	1/100
Anti-FcϵRI	FITC	AER-37	334608/Biolegend	1/100
Anti-TCR Vδ1	FITC	TS8.2	TCR2730/Invitrogen	1/100
Anti-CD57	FITC	HCD57	322306/Biolegend	1/50
Anti-NKp44	PercP/Cy5.5	P44-8	325114/Biolegend	2/50
Anti-TCR Vδ2	PercP/Cy5.5	B6	331423/Biolegend	1/100
Anti-DNAM-1	PercP/Cy5.5	11A8	338314/Biolegend	1/200
Anti-CD96	PE	NK92.39	338405/Biolegend	1/100
Anti-CD127	PE	A019D5	986002/Biolegend	1/100
Anti-TCR-V a24-Ja18	PE	6B11	342903/Biolegend	2/50
Anti-CD27	PE/Dazzle594	LG.7F9	302844/Biolegend	1/50
Anti-NKG2A	PE-Cy7	REA110	130-113-567/Miltenyi	1/200
Anti-CD39	PE-Cy7	A1	328212/Biolegend	1/50
Anti-CD94	APC	HP-3D9	559876/BD Pharmingen	1/100
Anti-CD4	APC	RPA-T4	300514/Biolegend	1/100
Anti-CD28	AF700	37.51	302920/Biolegend	1/400
Anti-CD45RA	AF700	HI100	560673/BD Pharmingen	1/100
Anti-CD137	AF700	4B4-1	309816/Biolegend	1/400
Anti-HLA-DR	APC-Cy7	1.243	307617/Biolegend	1/200
Anti-CD19	APC-Cy7	H1B19	302217/Biolegend	1/100
Anti-CD4	APC-Cy7	RPA-T4	300518/Biolegend	1/100
Anti-CD127	BV421	A019D5	351310/Biolegend	1/200
Anti-CD8	BV421	RPA-T8	30103/Biolegend	0.3/50
Anti-PD-1	BV421	EH12.2H7	301036/Biolegend	1/100
Anti-NKp46	BV510	9E2/NKp46	564064/BD Bioscience	1/100
Anti-CCR7	BV510	G043H7	35323/Biolegend	1/100
Anti-CD8	BV510	SK1	344732/Biolegend	1/100
Anti-CCR6	BV650	G034E3	353425/Biolegend	1/100
Anti-PD-1	BV650	EH12.2H7	564104/BD Bioscience	1/200
Anti-CD69	BV650	FN50	310934/Biolegend	1/200
Anti-c-kit	BV605	104D2	313218/Biolegend	1/200
Anti-CD25	BV605	BC96	302632/Biolegend	1/100
Anti-CD4	BV605	OKT4	317438/Biolegend	1/200
Anti-CXCR3	BV711	G025H7	353732/Biolegend	1/50
Anti-ICOS	BV711	15F9	313547/Biolegend	1/100
Anti-CD16	BV711	G18	302043/Biolegend	1/200
Anti-NKG2D	BV785	1D11	320829/Biolegend	1/50
Anti-TCRγδ	BV785	11F2	744743/BD Bioscience	1/50
Anti-CRTH2	BUV395	BM16	740319/BD Bioscience	4/50
Anti-CD3	BUV396	UCHT1	563546/BD Bioscience	1/100
Anti-CD56	BUV737	NCAM16.2	564447/BD Bioscience	1/800
Anti-CD69	BUV737	FN50	564439/BD Bioscience	1/100

**Table 2:** Demographics and baseline characteristics.

Variable	Categories	N (%)
Age	<60 y	6 (33.3)
	≥60 y	12 (66.6)
Gender	Female	9 (50)
	Male	9 (50)
ECOG PS	0	8 (44.4)
	1	9 (50)
	2	1 (5.5)
Smoking history	Current smoker	13 (72.2)
	Former smoker	2 (11.1)
	Non-smoker	3 (16.6)
Disease stage	IIIA	4 (22.2)
	IIIB	6 (33.3)
	IV	8 (44.4)
Tumor histology	Adenocarcinoma	5 (27.7)
	Squamous cell carcinoma	6 (33.3)
	NSCLC (NOS)	7 (38.8)
First-line treatment	Chemotherapy	2 (11.1)
	Radio + chemotherapy	16 (88.8)
Response to first-line treatment	CR	1 (5.5)
	PR	6 (33.3)
	SD	9 (50)
	PD	2 (11.1)
Other chemotherapy lines	Yes	14 (77.7)
	No	4 (22.2)

CR: complete response; PR: partial response; SD: stable disease; PD: progressive disease; NOS: not otherwise specified.

## Data Availability

The datasets generated in this study are available from the corresponding author on reasonable request. The data are not publicly available due to data are contained within the article.

## Supplementary Materials

The following supporting information can be downloaded at https://www.mdpi.com/article/10.3390/biomedicines13051122/s1. Figure S1: Changes in CD4+T cells population frequency during racotumomab-alum treatment in long-term (LS) and short-term survivors (SS); Figure S2: Changes in innate lymphocyte population frequency during racotumomab-alum treatment in long-term (LS) and short-term survivors (SS); Figure S3: Gate strategy for CD4+T cells, NK, NKT and γδT cells; Figure S4: Changes in innate lymphocyte population frequencies and ratio before and after racotumomab-alum treatment in long-term (LS) and short-term survivors (SS); Table S1: Values of frequencies (%) and cells ratio of immune populations represented in Figure 3; Table S2: Values of cytokines levels.

## Abbreviations

The following abbreviations are used in this manuscript:

NSCLC	Non-small cell lung cancer
MAbs	Monoclonal antibodies
TAA	Tumor-associated antigens
OS	Overall survival
PBMCs	Peripheral blood mononuclear cells
CM	Central memory
EM	Effector memory
TEMRA	Terminal effector memory cells
PS	Performance status
CR	Complete response
PR	Partial response
SD	Stable disease
PD	Progressive disease
NOS	Not otherwise specified
LS	Long-term survivors
SS	Short-term survivors
Tregs	Regulatory T cells
ICI	Checkpoints inhibitors
iNKT	Invariant NKT

## References

[1] Zhou G. (2019). Tobacco, air pollution, environmental carcinogenesis, and thoughts on conquering strategies of lung cancer. Cancer Biol. Med..

[2] Pineros M., Laversanne M., Barrios E., Cancela M.C., de Vries E., Pardo C., Bray F. (2022). An updated profile of the cancer burden, patterns and trends in Latin America and the Caribbean. Lancet Reg. Health Am..

[3] Chambers A., Routledge T., Pilling J., Scarci M. (2010). In elderly patients with lung cancer is resection justified in terms of morbidity, mortality and residual quality of life?. Interact. Cardiovasc. Thorac. Surg..

[4] Lahiri A., Maji A., Potdar P.D., Singh N., Parikh P., Bisht B., Mukherjee A., Paul M.K. (2023). Lung cancer immunotherapy: Progress, pitfalls, and promises. Mol. Cancer.

[5] Grimmett E., Al-Share B., Alkassab M.B., Zhou R.W., Desai A., Rahim M.M.A., Woldie I. (2022). Cancer vaccines: Past, present and future; a review article. Discov. Oncol..

[6] Shewell L.K., Wang J.J., Paton J.C., Paton A.W., Day C.J., Jennings M.P. (2018). Detection of N-glycolylneuraminic acid biomarkers in sera from patients with ovarian cancer using an engineered N-glycolylneuraminic acid-specific lectin SubB2M. Biochem. Biophys. Res. Commun..

[7] Hedlund M., Padler-Karavani V., Varki N.M., Varki A. (2008). Evidence for a human-specific mechanism for diet and antibody-mediated inflammation in carcinoma progression. Proc. Natl. Acad. Sci. USA.

[8] Tangvoranuntakul P., Gagneux P., Diaz S., Bardor M., Varki N., Varki A., Muchmore E. (2003). Human uptake and incorporation of an immunogenic nonhuman dietary sialic acid. Proc. Natl. Acad. Sci. USA.

[9] Samraj A.N., Laubli H., Varki N., Varki A. (2014). Involvement of a non-human sialic Acid in human cancer. Front. Oncol..

[10] Wang J., Shewell L.K., Day C.J., Jennings M.P. (2023). N-glycolylneuraminic acid as a carbohydrate cancer biomarker. Transl. Oncol..

[11] Vazquez A.M., Gabri M.R., Hernandez A.M., Alonso D.F., Beausoleil I., Gomez D.E., Perez R. (2000). Antitumor properties of an anti-idiotypic monoclonal antibody in relation to N-glycolyl-containing gangliosides. Oncol. Rep..

[12] Neninger E., Diaz R.M., de la Torre A., Rives R., Diaz A., Saurez G., Gabri M.R., Alonso D.F., Wilkinson B., Alfonso A.M. (2007). Active immunotherapy with 1E10 anti-idiotype vaccine in patients with small cell lung cancer: Report of a phase I trial. Cancer Biol. Ther..

[13] Diaz A., Alfonso M., Alonso R., Saurez G., Troche M., Catala M., Diaz R.M., Perez R., Vazquez A.M. (2003). Immune responses in breast cancer patients immunized with an anti-idiotype antibody mimicking NeuGc-containing gangliosides. Clin. Immunol..

[14] Alfonso S., Diaz R.M., de la Torre A., Santiesteban E., Aguirre F., Perez K., Rodriguez J.L., Barroso Mdel C., Hernandez A.M., Toledo D. (2007). 1E10 anti-idiotype vaccine in non-small cell lung cancer: Experience in stage IIIb/IV patients. Cancer Biol. Ther..

[15] Center for State Control of Medicines, E.a.M.D.C.I.H.C.f.S.C.o.M., Equipment and Medical Devices (CU); c2021. Registro. VAXIRA^®^ (Racotumomab). Reg. No.: B-013-001-L03C. https://www.cecmed.cu/registro/rcp/biologicos/vaxirar-racotumomab.

[16] Caceres-Lavernia H.H., Neninger-Vinageras E., Varona-Rodriguez L.M., Olivares-Romero Y.A., Sanchez-Rojas I., Mazorra-Herrera Z., Basanta-Bergolla D., Duvergel-Calderin D., Torres-Cuevas B.L., Castillo-Carrillo C. (2021). Racotumomab in Non-Small Cell Lung Cancer as Maintenance and Second-Line Treatment. MEDICC Rev..

[17] Sanchez L., Muchene L., Lorenzo-Luaces P., Viada C., Rodriguez P.C., Alfonso S., Crombet T., Neninger E., Shkedy Z., Lage A. (2018). Differential effects of two therapeutic cancer vaccines on short- and long-term survival populations among patients with advanced lung cancer. Semin. Oncol..

[18] Alfonso S., Valdes-Zayas A., Santiesteban E.R., Flores Y.I., Areces F., Hernandez M., Viada C.E., Mendoza I.C., Guerra P.P., Garcia E. (2014). A randomized, multicenter, placebo-controlled clinical trial of racotumomab-alum vaccine as switch maintenance therapy in advanced non-small cell lung cancer patients. Clin. Cancer Res..

[19] Van Damme V., Govaerts E., Nackaerts K., Dooms C., Wauters I., Vansteenkiste J. (2013). Clinical factors predictive of long-term survival in advanced non-small cell lung cancer. Lung Cancer.

[20] Hsu M.L., Murray J.C., Psoter K.J., Zhang J., Barasa D., Brahmer J.R., Ettinger D.S., Forde P.M., Hann C.L., Lam V.K. (2022). Clinical Features, Survival, and Burden of Toxicities in Survivors More Than One Year After Lung Cancer Immunotherapy. Oncologist.

[21] Horn L., Spigel D.R., Vokes E.E., Holgado E., Ready N., Steins M., Poddubskaya E., Borghaei H., Felip E., Paz-Ares L. (2017). Nivolumab Versus Docetaxel in Previously Treated Patients With Advanced Non-Small-Cell Lung Cancer: Two-Year Outcomes From Two Randomized, Open-Label, Phase III Trials (CheckMate 017 and CheckMate 057). J. Clin. Oncol..

[22] Herbst R.S., Garon E.B., Kim D.W., Cho B.C., Gervais R., Perez-Gracia J.L., Han J.Y., Majem M., Forster M.D., Monnet I. (2021). Five Year Survival Update From KEYNOTE-010: Pembrolizumab Versus Docetaxel for Previously Treated, Programmed Death-Ligand 1-Positive Advanced NSCLC. J. Thorac. Oncol..

[23] von Pawel J., Bordoni R., Satouchi M., Fehrenbacher L., Cobo M., Han J.Y., Hida T., Moro-Sibilot D., Conkling P., Gandara D.R. (2019). Long-term survival in patients with advanced non-small-cell lung cancer treated with atezolizumab versus docetaxel: Results from the randomised phase III OAK study. Eur. J. Cancer.

[24] Borghaei H., Gettinger S., Vokes E.E., Chow L.Q.M., Burgio M.A., de Castro Carpeno J., Pluzanski A., Arrieta O., Frontera O.A., Chiari R. (2021). Five-Year Outcomes From the Randomized, Phase III Trials CheckMate 017 and 057: Nivolumab Versus Docetaxel in Previously Treated Non-Small-Cell Lung Cancer. J. Clin. Oncol..

[25] Rodriguez P.C., Popa X., Martinez O., Mendoza S., Santiesteban E., Crespo T., Amador R.M., Fleytas R., Acosta S.C., Otero Y. (2016). A Phase III Clinical Trial of the Epidermal Growth Factor Vaccine CIMAvax-EGF as Switch Maintenance Therapy in Advanced Non-Small Cell Lung Cancer Patients. Clin. Cancer Res..

[26] Woo E.Y., Chu C.S., Goletz T.J., Schlienger K., Yeh H., Coukos G., Rubin S.C., Kaiser L.R., June C.H. (2001). Regulatory CD4^+^CD25^+^ T cells in tumors from patients with early-stage non-small cell lung cancer and late-stage ovarian cancer. Cancer Res..

[27] Yannelli J.R., Tucker J.A., Hidalgo G., Perkins S., Kryscio R., Hirschowitz E.A. (2009). Characteristics of PBMC obtained from leukapheresis products and tumor biopsies of patients with non-small cell lung cancer. Oncol. Rep..

[28] Li L., Chao Q.G., Ping L.Z., Xue C., Xia Z.Y., Qian D., Shi-ang H. (2009). The prevalence of FOXP3^+^ regulatory T-cells in peripheral blood of patients with NSCLC. Cancer Biother. Radiopharm..

[29] Koh J., Hur J.Y., Lee K.Y., Kim M.S., Heo J.Y., Ku B.M., Sun J.M., Lee S.H., Ahn J.S., Park K. (2020). Regulatory (FoxP3^+^) T cells and TGF-beta predict the response to anti-PD-1 immunotherapy in patients with non-small cell lung cancer. Sci. Rep..

[30] Borilova S., Grell P., Selingerova I., Gescheidtova L., Mlnarikova M., Bilek O., Lakomy R., Poprach A., Podhorec J., Kiss I. (2024). Early changes of peripheral circulating immune subsets induced by PD-1 inhibitors in patients with advanced malignant melanoma and non-small cell lung cancer. BMC Cancer.

[31] Li P., Qin P., Fu X., Zhang G., Yan X., Zhang M., Zhang X., Yang J., Wang H., Ma Z. (2021). Associations between peripheral blood lymphocyte subsets and clinical outcomes in patients with lung cancer treated with immune checkpoint inhibitor. Ann. Palliat. Med..

[32] Wang Y., Chen R., Guo Z., Wei W., Wang T., Ouyang R., Yuan X., Xing Y., Wang F., Wu S. (2024). Immunological profiling for short-term predictive analysis in PD-1/PD-L1 therapy for lung cancer. BMC Cancer.

[33] Teng M.W., Ngiow S.F., von Scheidt B., McLaughlin N., Sparwasser T., Smyth M.J. (2010). Conditional regulatory T-cell depletion releases adaptive immunity preventing carcinogenesis and suppressing established tumor growth. Cancer Res..

[34] Smyth M.J., Ngiow S.F., Teng M.W. (2014). Targeting regulatory T cells in tumor immunotherapy. Immunol. Cell Biol..

[35] van der Leun A.M., Thommen D.S., Schumacher T.N. (2020). CD8^+^ T cell states in human cancer: Insights from single-cell analysis. Nat. Rev. Cancer.

[36] Kim S., Koh J., Song S.G., Yim J., Kim M., Keam B., Kim Y.T., Kim J., Chung D.H., Jeon Y.K. (2022). High tumor hexokinase-2 expression promotes a pro-tumorigenic immune microenvironment by modulating CD8+/regulatory T-cell infiltration. BMC Cancer.

[37] Huang Y., Wu G., Bi G., Cheng L., Liang J., Li M., Zhang H., Shan G., Hu Z., Chen Z. (2024). Unveiling chemotherapy-induced immune landscape remodeling and metabolic reprogramming in lung adenocarcinoma by scRNA-sequencing. eLife.

[38] Geginat J., Lanzavecchia A., Sallusto F. (2003). Proliferation and differentiation potential of human CD8+ memory T-cell subsets in response to antigen or homeostatic cytokines. Blood.

[39] Manjarrez-Orduno N., Menard L.C., Kansal S., Fischer P., Kakrecha B., Jiang C., Cunningham M., Greenawalt D., Patel V., Yang M. (2018). Circulating T Cell Subpopulations Correlate With Immune Responses at the Tumor Site and Clinical Response to PD1 Inhibition in Non-Small Cell Lung Cancer. Front. Immunol..

[40] Larbi A., Fulop T. (2014). From “truly naive” to “exhausted senescent” T cells: When markers predict functionality. Cytom. Part A.

[41] Lee S.W., Choi H.Y., Lee G.W., Kim T., Cho H.J., Oh I.J., Song S.Y., Yang D.H., Cho J.H. (2021). CD8^+^ TILs in NSCLC differentiate into TEMRA via a bifurcated trajectory: Deciphering immunogenicity of tumor antigens. J. Immunother. Cancer.

[42] Crosby E.J., Hobeika A.C., Niedzwiecki D., Rushing C., Hsu D., Berglund P., Smith J., Osada T., Gwin Iii W.R., Hartman Z.C. (2020). Long-term survival of patients with stage III colon cancer treated with VRP-CEA(6D), an alphavirus vector that increases the CD8+ effector memory T cell to Treg ratio. J. Immunother. Cancer.

[43] Matsuda J.L., Mallevaey T., Scott-Browne J., Gapin L. (2008). CD1d-restricted iNKT cells, the ‘Swiss-Army knife’ of the immune system. Curr. Opin. Immunol..

[44] Tan J.Q., Xiao W., Wang L., He Y.L. (2010). Type I natural killer T cells: Naturally born for fighting. Acta Pharmacol. Sin..

[45] Krovi S.H., Gapin L. (2018). Invariant Natural Killer T Cell Subsets-More Than Just Developmental Intermediates. Front. Immunol..

[46] Motohashi S., Kobayashi S., Ito T., Magara K.K., Mikuni O., Kamada N., Iizasa T., Nakayama T., Fujisawa T., Taniguchi M. (2002). Preserved IFN-alpha production of circulating Valpha24 NKT cells in primary lung cancer patients. Int. J. Cancer.

[47] Bricard G., Cesson V., Devevre E., Bouzourene H., Barbey C., Rufer N., Im J.S., Alves P.M., Martinet O., Halkic N. (2009). Enrichment of human CD4^+^ Vα24/Vβ11 invariant NKT cells in intrahepatic malignant tumors. J. Immunol..

[48] Tachibana T., Onodera H., Tsuruyama T., Mori A., Nagayama S., Hiai H., Imamura M. (2005). Increased intratumor Valpha24-positive natural killer T cells: A prognostic factor for primary colorectal carcinomas. Clin. Cancer Res..

[49] Tahir S.M., Cheng O., Shaulov A., Koezuka Y., Bubley G.J., Wilson S.B., Balk S.P., Exley M.A. (2001). Loss of IFN-gamma production by invariant NK T cells in advanced cancer. J. Immunol..

[50] Molling J.W., Kolgen W., van der Vliet H.J., Boomsma M.F., Kruizenga H., Smorenburg C.H., Molenkamp B.G., Langendijk J.A., Leemans C.R., von Blomberg B.M. (2005). Peripheral blood IFN-gamma-secreting Vα24^+^Vβ11^+^ NKT cell numbers are decreased in cancer patients independent of tumor type or tumor load. Int. J. Cancer.

[51] Gentilini M.V., Perez M.E., Fernandez P.M., Fainboim L., Arana E. (2016). The tumor antigen N-glycolyl-GM3 is a human CD1d ligand capable of mediating B cell and natural killer T cell interaction. Cancer Immunol. Immunother..

[52] Dockry E., O’Leary S., Gleeson L.E., Lyons J., Keane J., Gray S.G., Doherty D.G. (2018). Epigenetic induction of CD1d expression primes lung cancer cells for killing by invariant natural killer T cells. Oncoimmunology.

[53] Sanmamed M.F., Perez-Gracia J.L., Schalper K.A., Fusco J.P., Gonzalez A., Rodriguez-Ruiz M.E., Onate C., Perez G., Alfaro C., Martin-Algarra S. (2017). Changes in serum interleukin-8 (IL-8) levels reflect and predict response to anti-PD-1 treatment in melanoma and non-small-cell lung cancer patients. Ann. Oncol..

[54] Okuma Y., Wakui H., Utsumi H., Sagawa Y., Hosomi Y., Kuwano K., Homma S. (2018). Soluble Programmed Cell Death Ligand 1 as a Novel Biomarker for Nivolumab Therapy for Non-Small-cell Lung Cancer. Clin. Lung. Cancer.

[55] Costantini A., Julie C., Dumenil C., Helias-Rodzewicz Z., Tisserand J., Dumoulin J., Giraud V., Labrune S., Chinet T., Emile J.F. (2018). Predictive role of plasmatic biomarkers in advanced non-small cell lung cancer treated by nivolumab. Oncoimmunology.

[56] Xu D.H., Zhu Z., Wakefield M.R., Xiao H., Bai Q., Fang Y. (2016). The role of IL-11 in immunity and cancer. Cancer Lett..

[57] Leung J.H., Ng B., Lim W.W. (2022). Interleukin-11: A Potential Biomarker and Molecular Therapeutic Target in Non-Small Cell Lung Cancer. Cells.

[58] Tao L., Huang G., Wang R., Pan Y., He Z., Chu X., Song H., Chen L. (2016). Cancer-associated fibroblasts treated with cisplatin facilitates chemoresistance of lung adenocarcinoma through IL-11/IL-11R/STAT3 signaling pathway. Sci. Rep..

[59] Langrish C.L., McKenzie B.S., Wilson N.J., de Waal Malefyt R., Kastelein R.A., Cua D.J. (2004). IL-12 and IL-23: Master regulators of innate and adaptive immunity. Immunol. Rev..

[60] Gaffen S.L., Jain R., Garg A.V., Cua D.J. (2014). The IL-23-IL-17 immune axis: From mechanisms to therapeutic testing. Nat. Rev. Immunol..

[61] Yan J., Smyth M.J., Teng M.W.L. (2018). Interleukin (IL)-12 and IL-23 and Their Conflicting Roles in Cancer. Cold Spring Harb. Perspect. Biol..

[62] Liu D., Xing S., Wang W., Huang X., Lin H., Chen Y., Lan K., Chen L., Luo F., Qin S. (2020). Prognostic value of serum soluble interleukin-23 receptor and related T-helper 17 cell cytokines in non-small cell lung carcinoma. Cancer Sci..

[63] Yang K., Tian C., Zhang C., Xiang M. (2022). The Controversial Role of IL-33 in Lung Cancer. Front. Immunol..

[64] Feng Y., Zhu Y., Luo G., Wang Z., Yu P., Zheng L. (2016). Expression and clinical significance of IL-33 in patients with non-small cell lung cancer. Xi Bao Yu Fen Zi Mian Yi Xue Za Zhi.

[65] Li A., Herbst R.H., Canner D., Schenkel J.M., Smith O.C., Kim J.Y., Hillman M., Bhutkar A., Cuoco M.S., Rappazzo C.G. (2019). IL-33 Signaling Alters Regulatory T Cell Diversity in Support of Tumor Development. Cell Rep..

[66] Wen Y.H., Lin H.Q., Li H., Zhao Y., Lui V.W.Y., Chen L., Wu X.M., Sun W., Wen W.P. (2019). Stromal interleukin-33 promotes regulatory T cell-mediated immunosuppression in head and neck squamous cell carcinoma and correlates with poor prognosis. Cancer Immunol. Immunother..

