# Supplementary material for: Treatment of Advanced NSCLC Patients with an Anti-Idiotypic NeuGcGM3-Based Vaccine: Immune Correlates in Long-Term Survivors
Source: Biomedicines. 2025 May 6;13(5):1122. doi: 10.3390/biomedicines13051122 (PMC12109512; doi:10.3390/biomedicines13051122)
Supplement: Supplementary file 1 [file biomedicines-13-01122-s001.zip › Figure S2 innate.pdf]

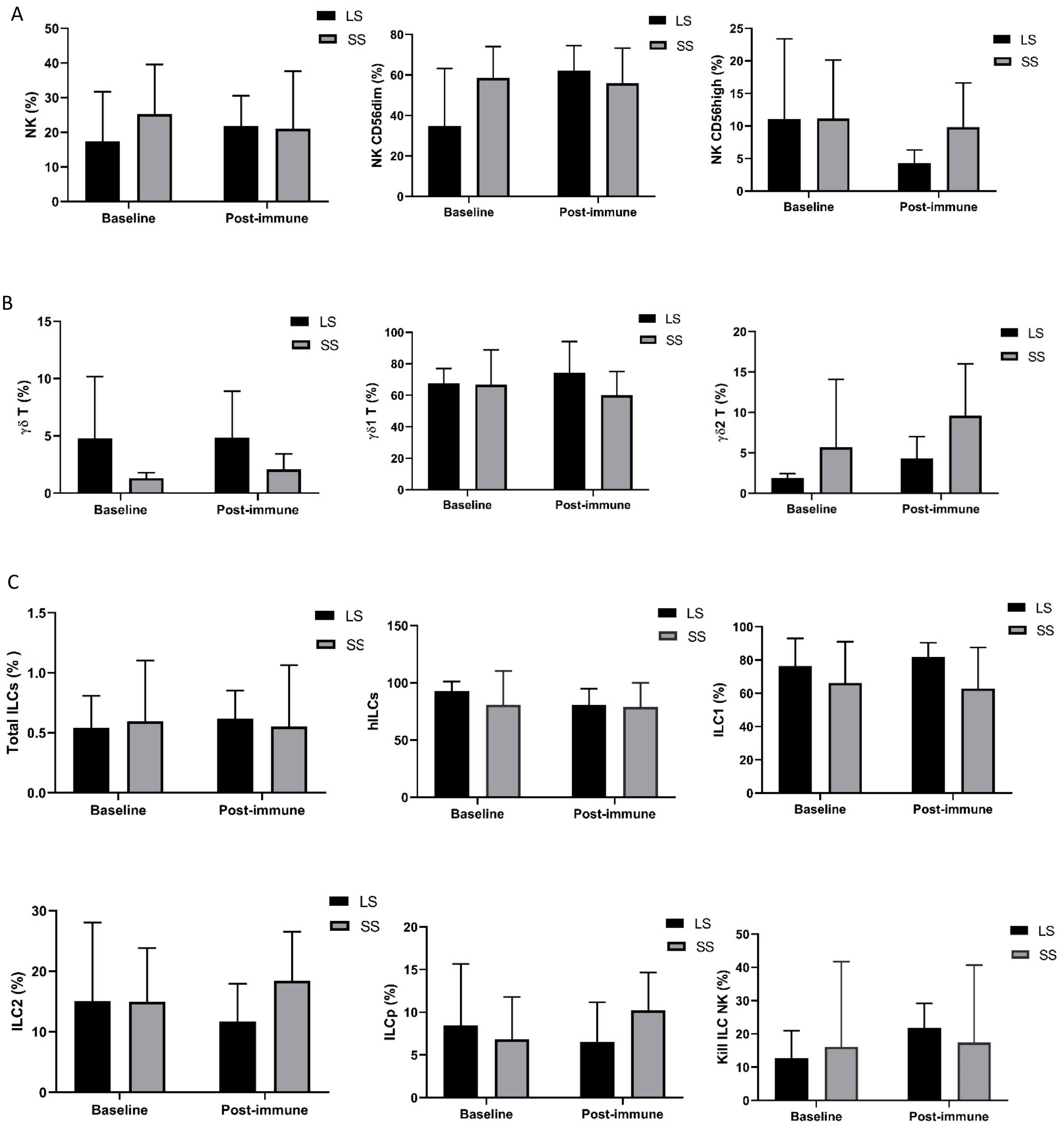

Figure S2: Changes of innate lymphocyte populations frequency during racotumomab-alum treatment in long-term (LS) and short-term survivors (SS). A) Behavior of NK cells, B) γδ T cells and C) ILC and subpopulations. No significant differences among the groups were found ($p > 0.05$, Mann-Whitney U test).
