# Supplementary material for: Treatment of Advanced NSCLC Patients with an Anti-Idiotypic NeuGcGM3-Based Vaccine: Immune Correlates in Long-Term Survivors
Source: Biomedicines. 2025 May 6;13(5):1122. doi: 10.3390/biomedicines13051122 (PMC12109512; doi:10.3390/biomedicines13051122)
Supplement: Supplementary file 1 [file biomedicines-13-01122-s001.zip › Figure S4 pre and post.pdf]

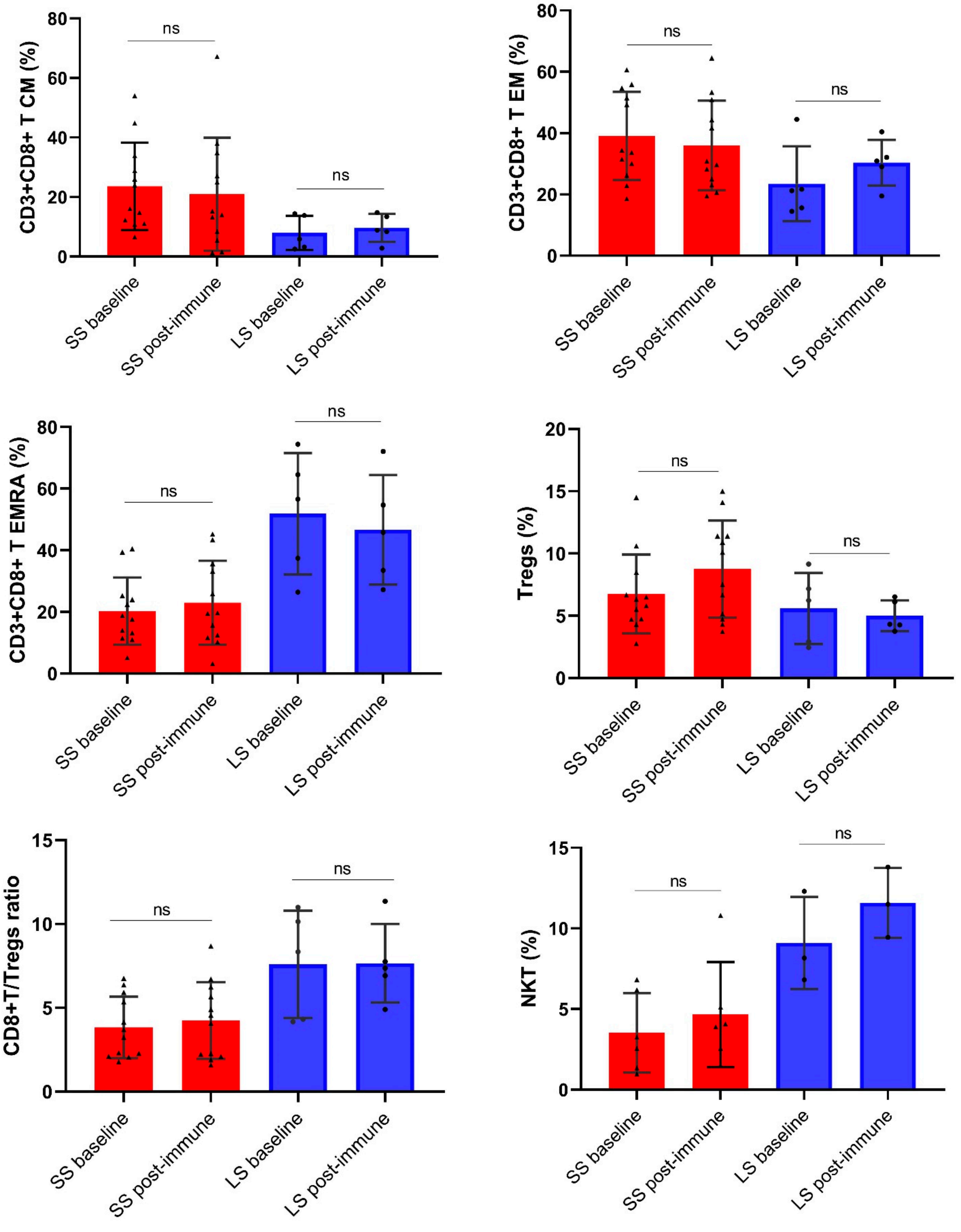

Figure S4: Changes of innate lymphocyte populations frequencies and ratio before and after racotumomab-alum treatment in long-term (LS) and short-term survivors (SS). No significant differences between baseline

and post-immune samples for both groups were found ($p > 0.05$, Wilcoxon Signed-Ranks Test).
