# Supplementary material for: Treatment of Advanced NSCLC Patients with an Anti-Idiotypic NeuGcGM3-Based Vaccine: Immune Correlates in Long-Term Survivors
Source: Biomedicines. 2025 May 6;13(5):1122. doi: 10.3390/biomedicines13051122 (PMC12109512; doi:10.3390/biomedicines13051122)
Supplement: Supplementary file 1 [file biomedicines-13-01122-s001.zip › Table S1.pdf]

Table S1. Values of frequencies (%) and cells ratio of immune populations represented in Figure 3

Patients	Immune populations (%)										Immune populations ratio	
	CD8 CM (BS)	CD8 CM (PI)	CD8 EM (BS)	CD8 EM (PI)	CD8 EMRA (BS)	CD8 EMRA (PI)	Tregs (BS)	Tregs (PI)	NKT (BS)	NKT (PI)	CD8/Tregs (BS)	CD8/Tregs (PI)
LS1	13.8	2.86	15.6	19.5	74.4	72.1	7.18	6.12	6.82	9.46	4.17	6.92
LS2	14.4	13.4	21.2	29	37.4	33.5	2.93	4.28	8.17	11.5	4.32	4.91
LS3	5.9	8.93	44.5	40.4	26.4	54.7	2.45	4.33	12.3	13.8	10.1	7.75
LS4	2.57	8.35	21.7	32.1	64.6	45.8	6.24	3.76	NE	NE	11	11.36
LS5	3.26	14.8	14.5	30.9	56.6	27.2	9.16	6.52	NE	NE	8.34	7.37
SS1	14.8	15.3	49.3	41.7	24	19.7	4.76	7.53	2.57	5.12	6.75	4.56
SS2	12.3	13.3	60.6	20.6	14	33.2	6.67	10.9	0.98	1.5	6.37	1.89
SS3	11.1	14	18.6	28.3	39.4	25.9	2.78	10.1	6.19	2.57	2.06	2.09
SS4	6.64	5.52	51.5	64.5	18.8	12.5	5.52	3.74	3.27	4.09	5.92	8.68
SS5	33.9	38	54.7	53.3	11	11.7	8.49	5.16	6.83	3.9	4.14	4.9
SS6	44.8	34.9	34.4	29.7	11.5	15.7	6.34	15	1.35	10.8	2.28	2.27
SS7	54	67.2	55.9	23.1	5.18	3.23	6.51	4.33	NE	NE	5.36	6.24
SS8	10.8	1.28	33.7	50.9	25.3	43.4	10.6	11.4	NE	NE	2.32	5.66
SS9	16.1	27.1	26.2	25	40.5	19.5	4.31	6.67	NE	NE	3.24	1.59
SS10	28.9	25	31.5	44.3	22.4	10.2	5.8	4.73	NE	NE	1.78	2.23
SS11	26	8.53	22.9	30.9	17.6	45.2	14.5	14.1	NE	NE	3.7	4.08
SS12	24	1.56	30.1	19.5	13.2	35.7	4.73	11.4	NE	NE	2.1	6.71

LS: Long-term survivors; SS: Short-term survivors; NE: non-evaluated; BS: baseline; PI: post-immune
