# Supplementary material for: Treatment of Advanced NSCLC Patients with an Anti-Idiotypic NeuGcGM3-Based Vaccine: Immune Correlates in Long-Term Survivors
Source: Biomedicines. 2025 May 6;13(5):1122. doi: 10.3390/biomedicines13051122 (PMC12109512; doi:10.3390/biomedicines13051122)
Supplement: Supplementary file 1 [file biomedicines-13-01122-s001.zip › Table S2.pdf]

Table S2. Values of cytokines levels

Patients	Cytokines (pg/ml)					
	IL-11 (BS)	IL-11 (PI)	IL-23 (BS)	IL-23 (PI)	IL-33 (BS)	IL-33 (PI)
LS1	0	224	15	167	0	0
LS2	0	563	0	1433	0	96
LS3	0	0	0	45	0	0
LS4	39.5	96	0	1366	40	0
LS5	0	896	0	1602	0	43
SS1	0	4070	0	3959	0	468
SS2	0	2346	0	1615	0	479
SS3	0	6624	0	2805	0	1236
SS4	0	10534	10	4906	0	200
SS5	0	27401	0	5109	0	305

LS: Long-term survivors; SS: Short-term survivors; BS: baseline; PI: post-immune
