# Supplementary figures and images for: Treatment of Advanced NSCLC Patients with an Anti-Idiotypic NeuGcGM3-Based Vaccine: Immune Correlates in Long-Term Survivors
Source: Biomedicines. 2025 May 6;13(5):1122. doi: 10.3390/biomedicines13051122 (PMC12109512; doi:10.3390/biomedicines13051122)

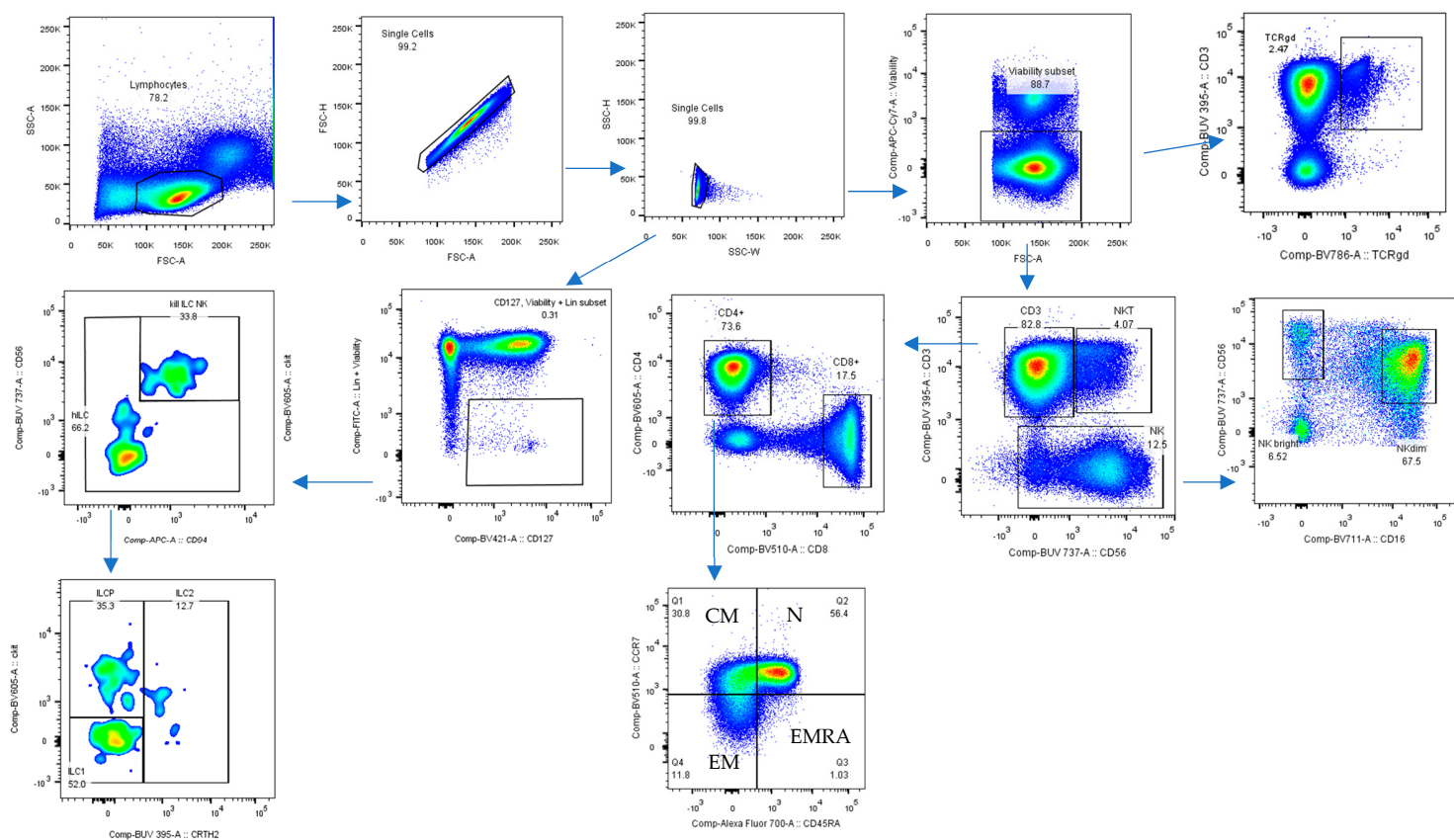

Figure S3. Gate strategy for CD4+T cells, NK, NKT and  $\gamma\delta$ T cells.

Supplement: Supplementary file 1 [file biomedicines-13-01122-s001.zip › Figure S3 gate strategies.pdf]
